# Innovative antifungal strategies: enhanced biofilm inhibition of *Candida albicans* by a modified tea tree oil formulation

**DOI:** 10.3389/fmicb.2024.1518598

**Published:** 2025-01-15

**Authors:** Dang Anh Tuan, Pham Vu Nhat Uyen, Nguyen Van Khuon, Ly An Binh, Jan Masak

**Affiliations:** ^1^Faculty of Food and Biochemical Technology, University of Chemistry and Technology Prague, Prague, Czechia; ^2^Faculty of Chemical Engineering, Industrial University of Ho Chi Minh City, Ho Chi Minh City, Vietnam; ^3^Public Health Station, Ho Chi Minh City, Vietnam; ^4^An Binh Hospital, Ho Chi Minh City, Vietnam

**Keywords:** *Candida albicans*, tea tree oil, biofilm, antifungal, biofilm inhibition, modified tea tree oil formulation, Minimum Fungicidal Concentration (MFC), Minimum Biofilm Inhibitory Concentration (MBIC)

## Abstract

**Introduction:**

*Candida albicans* is a significant human pathogen with the ability to form biofilms, a critical factor in its resistance to antifungal treatments. This study aims to evaluate the antifungal activity and biofilm inhibition potential of Tea Tree Oil (TTO) derived from *Melaleuca alternifolia* cultivated in Vietnam.

**Methods:**

The antifungal activity of TTO was assessed by determining the Minimum Inhibitory Concentration (MIC), Minimum Fungicidal Concentration (MFC), Minimum Biofilm Inhibitory Concentration (MBIC), and Minimum Biofilm Eradication Concentration (MBEC) using broth dilution methods. The experiments were conducted on *C. albicans* in both planktonic and biofilm states across concentrations ranging from 0.1 μL/mL to 10 μL/mL.

**Results:**

TTO demonstrated significant antifungal efficacy, with a MIC of 0.1 μL/mL (∼91.217 μg/mL) and an MFC of 10 μL/mL (∼9121.7 μg/mL). It effectively inhibited biofilm formation with a recorded MBIC of 2 μL/mL (∼1824.34 μg/mL). However, MBEC values were not determinable as the concentrations tested did not achieve the eradication of more than 50% of mature biofilm within the experimental conditions.

**Discussion:**

These findings highlight TTO as a promising natural antifungal agent with strong biofilm-inhibitory properties. However, its limited efficacy in eradicating mature biofilms underscores the need for further studies, potentially involving higher concentrations or synergistic combinations with conventional antifungal agents.

## 1 Introduction

*Candida albicans* is widely prevalent in the human microbiota and possesses opportunistic pathogenic traits, contributing to the development of serious, sometimes fatal, bloodstream infections (Lohse et al., 2018). The crucial virulence factor in its pathogenesis lies in the initiation of adhesion and the subsequent formation of biofilms on both abiotic and biotic surfaces (Figueiral et al., 2007). The growing apprehension about drug resistance emphasizes the urgency of discovering new agents, leading to an increased focus on exploring novel antifungal compounds effective against biofilms (Grando et al., 2016). Traditional medicinal plants and alternative therapies have emerged as promising sources for identifying innovative antimicrobial agents.

Natural products play a crucial role in the exploration of novel antifungal therapies. Numerous studies have underscored the antimicrobial effectiveness of these products against oral pathogens, positioning herbal agents as potential tools for preventing and managing infectious oral diseases (Catalán et al., 2008; Hammer et al., 2003b). In Vietnam, *Melaleuca alternifolia*, a member of the Myrtaceae family native to Australia commonly known as tea tree, produces an essential oil known as tea tree oil (TTO) through the steam distillation of its leaves. Containing around 100 compounds, among which terpinen-4-ol constitutes a minimum of 30% of the oil, and with notable presence of 1,8-cineole and terpinolene, TTO displays a diverse array of biological activities. These actions aid in efficiently handling diverse microorganisms, improving approaches to tackle persistent infections such as candidiasis, pharyngitis, vaginitis, and respiratory tract diseases. TTO influences biological membranes by compromising their integrity and impeding enzyme function, leading to heightened membrane fluidity and the release of intracellular components (Cox and Markham, 2007). As highlighted by Cox and Markham (2007), the hydrophobic nature of its chemical constituents enables them to accumulate in lipid-rich membrane environments, resulting in structural and functional damage.

Carson et al. (2006) conducted a study that determined the concentration ranges for key terpenes, alcohols, and ethers in TTO. The study also provided guidelines stating that the terpinen-4-ol content should be a minimum of 30% and the 1,8-cineole content should not exceed 15% of the oil volume. Due to its antimicrobial, antifungal, and anti-inflammatory properties, terpinen-4-ol, which is the main active component in TTO, has attracted significant attention (Francisconi et al., 2015). Notably, terpinen-4-ol induces membrane disruption, compromising the integrity and physiology of microbial cells (Carson et al., 2006; Maquera Huacho et al., 2019; Maquera-Huacho et al., 2018; Ramage et al., 2012). By virtue of its low concentrations, it demonstrates non-toxicity toward fibroblasts and epithelial cells, making it suitable for topical use with minimal adverse effects.

In this study, the chemical composition of Tea Tree Oil (TTO) was analyzed using gas chromatography-mass spectrometry (GC-MS), identifying a range of bioactive compounds. The major components include **eucalyptol (18-cineole)**, which constitutes 29.41% of the oil. This compound plays a significant role in enhancing the permeability of microbial cell membranes, facilitating the penetration of other compounds and disrupting internal cellular processes (Brun et al., 2019). **Terpinolene**, accounting for 8.63%, is another major compound known for its strong antimicrobial and antifungal properties, disrupting the cell membranes of *C. albicans* and inhibiting fungal growth (Carson et al., 2006). Additionally, **γ -terpinene** (7.92%) has antioxidant and antimicrobial properties that further contribute to the disruption of microbial cell structures (Maquera Huacho et al., 2019), while **α -terpineol** (5.67%) has been shown to effectively inhibit the growth of *C. albicans* and other bacteria (Hammer et al., 2003a). Furthermore, the most bioactive component, **terpinen-4-ol**, is known for its membrane-disrupting effects on *C. albicans*, altering membrane permeability and causing ion imbalance, which leads to microbial cell death (Ramage et al., 2012). These findings highlight the complex chemical profile of TTO and its potent antifungal and biofilm-inhibitory properties, supporting its use as an effective agent against *C. albicans*.

This study aims to evaluate the antifungal and biofilm-inhibitory potential of a modified formulation of TTO derived from *Melaleuca alternifolia* cultivated in Vietnam. We investigate its MIC (Minimum Inhibitory Concentration, the lowest concentration of a substance that inhibits visible growth of a microorganism), MFC (Minimum Fungicidal Concentration, the lowest concentration of a substance that kills ≥99.9% (or a 3 Log reduction) of the fungal population), MBIC (Minimum Biofilm Inhibitory Concentration, the lowest concentration of an antifungal that prevents biofilm formation), and MBEC (Minimum Biofilm Eradication Concentration, the lowest concentration of an antifungal that eradicates pre-formed biofilm by ≥50%) against *C. albicans*. By exploring both planktonic and biofilm states of *C. albicans*, this study provides a comprehensive evaluation of TTO’s potential as a novel therapeutic agent for managing fungal infections, particularly those complicated by biofilm formation. Additionally, we highlight the novelty of using a modified TTO formulation with enhanced concentrations of its key bioactive components to assess its efficacy compared to previously studied formulations.

## 2 Materials and methods

### 2.1 Microorganisms, cultivation environment, and conditions

A laboratory intervention experiment was conducted at the Faculty of Chemical Engineering, Ho Chi Minh City University of Technology (HCMUT), Vietnam National University-Ho Chi Minh City (VNU-HCMC). The microbial strain under investigation was the *C. albicans* strain (ATCC 24433) purchased from KwikStik Co., Ltd, Vietnam. They were activated and cultured in a self-prepared liquid Hansen medium (comprising 50 g H_2_SO_4_, 3 g KH_2_PO_4_, 3 g MgSO_4_.7H_2_O, 10 g peptone, 20 g agar, 1000 mL distilled water, adjusted to pH 6), at room temperature, aerobic conditions, and shaken at 150 rpm for 24 h. The Hansen agar medium used for *C. albicans* growth had the following composition: 50 g/L H_2_SO_4_, 3 g/L KH_2_PO_4_, 3 g/L MgSO_4_⋅7H_2_O, 10 g/L peptone, 20 g/L agar, and 1 L distilled water. The pH was adjusted to 6.0 before autoclaving at 121°C for 15 min, as per standard protocols (Rodriguez-Tudela et al., 2001; Silva et al., 2009; Vaňková et al., 2020).

### 2.2 Bioactive compounds

This study utilized pure tea tree oil (*Melaleuca alternifolia*) obtained from Thuy Moc Viet Co., Ltd, Vietnam. The chemical components of the tea tree oil were analyzed by the Institute of Materials Science - Vietnam Academy of Science and Technology. Additionally, the antifungal agent Amphotericin B (AMB) was purchased from Maxxcare VP Co., Ltd, India, and used as a positive control in antifungal activity assays and biofilm inhibition studies against *C. albicans*.

### 2.3 Rationale for using volume-based units

Essential oils are complex mixtures of volatile and hydrophobic compounds, and their precise molecular composition can vary depending on factors such as the source plant, extraction methods, and storage conditions. Given this variability, their antifungal activity is conventionally expressed in volume-based units (e.g., μL/mL) in research to ensure practical standardization and reproducibility across studies. This approach aligns with established protocols for essential oils, which prioritize biological activity over exact chemical composition. However, for consistency when comparing essential oils with conventional drugs, which are typically expressed in weight-based units, weight equivalents for TTO concentrations are provided in this study (e.g., μg/mL).

### 2.4 Preparation of emulsions

Due to the hydrophobic nature of tea tree oil (TTO), it was emulsified with sterile distilled water containing 0.5% Tween 80 before being mixed into the culture medium, following the method described by Van Nguyen (2017). However, some adjustments were made to ensure compatibility with experimental conditions, with final concentrations achieved after dilution being 0.1, 1, 2, 5, and 10 μL/mL (equivalent to approximately 91.217, 912.17, 1824.34, 4560.85, and 9121.7 μg/mL, respectively). The entire experiment was conducted at these five concentration thresholds. The AMB antifungal agent used as a positive control was also dissolved in sterile distilled water containing 0.5% Tween 80 and volumes were calculated to achieve final concentrations of 0.1, 1, 2, 5, and 10 μg/mL.

### 2.5 Selection of culture medium

The Mueller-Hinton medium, supplemented with 0.5% Tween 80, was chosen over RPMI-1640 due to its enhanced compatibility with hydrophobic substances like TTO. This combination ensures better solubility and diffusion of the essential oil in the aqueous medium, which is critical for accurate determination of the Minimum Inhibitory Concentration (MIC) and Minimum Fungicidal Concentration (MFC). While RPMI-1640 is commonly used in antifungal testing, the choice of Mueller-Hinton was based on its success in studies with essential oils, ensuring consistent and reproducible results under the specific conditions of this research (Chen et al., 2023; Hulankova, 2022; Otajevwo and Osawaru, 2020).

### 2.6 Determining the Minimum Inhibitory Concentration (MIC) and Minimum Fungicidal Concentration (MFC)

For MIC determination, *C. albicans* was first cultured in Hansen medium, incubated at room temperature with shaking at 150 rpm for 24 h. The cells were centrifuged at 4000 rpm for 20 min, washed with PBS, and resuspended in Mueller-Hinton medium. Cell density was adjusted to an OD range of 0.119–0.14 at 530 nm (approximately 1 × 10^6^–6.2 × 10^6^ CFU/mL). In the test tubes, 4 mL of Mueller-Hinton medium containing the cell suspension was mixed with 1 mL of emulsified TTO, yielding final concentrations of 0.1, 1, 2, 5, and 10 μL/mL (equivalent to approximately 91.217, 912.17, 1824.34, 4560.85, and 9121.7 μg/mL, respectively). Controls included AMB (0.1–10 μg/mL) and untreated Mueller-Hinton medium. After a 24-h incubation at room temperature, the optical density was measured at 610 nm using a UV-VIS spectrophotometer to determine the MIC, defined as the lowest concentration that inhibited fungal growth compared to controls (Casagrande Pierantoni et al., 2019; Makambi et al., 2023; Rouf et al., 2017; Xie et al., 2012).

The Minimum Fungicidal Concentration (MFC) was determined by subculturing 50 μL aliquots from test tubes showing no visible fungal growth onto fresh Hansen agar plates and incubating them for 48 h at room temperature. The MFC was defined as the lowest concentration of TTO at which no fungal growth was observed on the agar plates. This protocol is based on the method described by Rodriguez-Tudela et al. (2001), which has been widely utilized in antifungal research. While this method aligns with established fungal studies, it deviates from the ≥99.9% reduction criterion typically applied in bactericidal assays, as no universal standard exists for fungal MFC determination. The subculturing volume (50 μL) was chosen to ensure sufficient sensitivity while maintaining consistency with validated protocols for fungal susceptibility testing.

MIC represents the lowest concentration inhibiting visible fungal growth, while MIC_80_ is the concentration causing an 80% reduction in optical density compared to the control. MFC is defined as the lowest concentration showing no colony formation on agar after 48 h (Makambi et al., 2023; Xie et al., 2012).

Following that, 4 mL of Mueller-Hinton medium containing fungal cell suspension was added to each test tube. Then, 1 mL of TTO, emulsified with sterile distilled water containing 0.5% Tween 80, was added to achieve final concentrations of 0.1, 1, 2, 5, 10 μL/mL (equivalent to approximately 91.217, 912.17, 1824.34, 4560.85, and 9121.7 μg/mL, respectively) in the medium. The positive control (AMB) consisted of 4 mL of Mueller-Hinton medium containing fungal cell suspension and 1 mL of AMB mixed in sterile distilled water containing 0.5% Tween 80, resulting in final concentrations of 0.1, 1, 2, 5, 10 μg/mL. The negative control contained 4 mL of Mueller-Hinton medium with fungal cell suspension and 1 mL of sterile distilled water containing 0.5% Tween 80. Blank samples were prepared similarly but without fungal cell suspension. The tubes were then incubated at room temperature for 24 h. After incubation, the optical density was measured at a wavelength of 610 nm using a UV-VIS spectrophotometer to determine the MIC of TTO in inhibiting fungal growth.

From the tubes identified as MICs, 50 μL of these samples were spread onto the surface of Hansen medium and incubated at room temperature for 48 h to determine the MFC of TTO. The MIC and MFC experiments were conducted independently three times, and the averages were calculated.

MIC is defined as the lowest concentration of TTO or antifungal agent that inhibits the fungal growth, as indicated by a reduction in optical density compared to the control sample (Vaňková et al., 2020; Vu et al., 2023). MIC_80_ is the lowest concentration of tea tree oil or antifungal agent causing an 80% reduction in optical density compared to the control sample (Vaňková et al., 2020; Vu et al., 2023). MFC is the lowest concentration of TTO or antifungal agent demonstrating no growth of fungal colonies on the agar surface after 48 h of incubation (Blanc et al., 2023; Salem et al., 2018).

### 2.7 Investigation of the biofilm-forming ability of *C. albicans*

*C. albicans* strains were cultured in Hansen medium with additional glucose concentrations of 2.5, 5, and 10% (w/v) in 2 mL round-bottom Eppendorf tubes.

Materials preparation followed Pereira et al. (2015) with minor adjustments. *C. albicans* was cultured on agar for 48 h, then inoculated into Hansen liquid medium at room temperature for 24 h with shaking at 150 rpm. After centrifugation at 4000 rpm for 20 min and two PBS washes, the pellet was resuspended in Hansen medium with glucose concentrations of 2.5, 5, and 10% (w/v). The cell density was adjusted to approximately 10^7^ CFU/mL [OD_530__*nm*_ values between 0.39 and 0.43, equivalent to 1.26 × 10^7^–6.49 × 10^7^ CFU/mL] (Rodriguez-Tudela et al., 2001). Subsequently, 1 mL of the standard inoculum [yeast cell suspension in Hansen medium with 2.5, 5, and 10% glucose (w/v)] was aspirated into 2 mL Eppendorf tubes and incubated undisturbed at room temperature for 24 h.

Biofilm formation capability was assessed by quantifying total biomass using the Crystal Violet (CV) staining method, adapted from Silva et al. (2009). After a 24-h incubation, wells were washed with sterile PBS (pH 7.2) three times to remove residual medium and non-adherent fungal cells. Methanol (1 mL) was added and removed after 15 min. Eppendorf tubes were air-dried, followed by the addition of 1 mL CV 1% (v/v) for 5 min. After washing with PBS, 33% (v/v) acetic acid (1 mL) was added, and absorbance was read at 570 nm using a UV-VIS spectrophotometer. The experiment was conducted in triplicate, with three parallel samples for each repetition.

In this study, we opted to use 2 mL round-bottom Eppendorf microtubes instead of 96-well microplates to grow *C. albicans* biofilms. Microtubes provide a more enclosed environment, which helps prevent medium evaporation and ensures stable conditions for biofilm formation during long-term experiments, especially under static conditions (Stewart and Franklin, 2008). Additionally, microtubes allow for more accurate control of aeration and mixing when placed on a shaker, facilitating better biofilm formation by reducing the risk of desiccation or inconsistent nutrient diffusion. Microtubes also simplify handling during the washing and staining steps required for biofilm quantification assays, enhancing reproducibility due to the consistent geometry of the tubes (Merritt et al., 2011).

Biofilm-forming ability, assessed by total biomass quantification, followed Alves et al.’s (2023) method:


O⁢D⁢c=O⁢D⁢n⁢c+3×S⁢D

Where:

ODc: The cut-off optical density

ODnc: The mean OD of the negative control

SD: The standard deviations of OD values of negative control samples (ODs)

Positive samples (ODs > ODc) were considered biofilm producers, categorized as:

ODs ≤ ODc: no biofilm producer

ODc < ODs ≤ 2 × ODc: week biofilm producer

2 × ODc < ODs ≤ 4 × ODc: moderate biofilm producer

4 × ODc < ODs: strong biofilm producer

### 2.8 Determining the Minimum Biofilm Inhibitory Concentration (MBIC) and Minimum Biofilm Eradication Concentration (MBEC)

The cell density in the *C. albicans* yeast suspension for MBIC and MBEC determination should be around 10^7^ CFU/mL, [OD_530 nm_ values between 0.39 and 0.43, approximately 1.26 × 10^7^–6.49 × 10^7^ CFU/mL] (Rodriguez-Tudela et al., 2001) and must be re-suspended in Hansen medium (the Hansen medium composition was selected from the *C. albicans* biofilm formation capability assessment experiment).

MBIC and MBEC are defined as the lowest concentration of essential oil that inhibits over 50% of biofilm formation and the lowest concentration of essential oil that eradicates over 50% of pre-formed biofilm, respectively (Purwasena et al., 2020).

MBIC and MBEC were determined following Purwasena et al.’s (2020) method, with adjustments. *C. albicans* yeast cell suspension (900 μL) in 2 mL Eppendorf tubes was prepared. Different concentrations of essential oil (0.1, 1, 2, 5, and 10 μL/mL equivalent to approximately 91.217, 912.17, 1824.34, 4560.85, and 9121.7 μg/mL, respectively) were added in 100 μL increments. Positive controls (using AMB) were also prepared at concentrations of 0.1, 1, 2, 5, and 10 μg/mL. The tubes were statically incubated for 24 h at room temperature. After incubation, the medium was removed, and tubes were washed with sterile PBS buffer (pH 7.2) three times to eliminate free cells. Biofilms were stained with 0.1% crystal violet for 30 min. After staining, crystal violet was removed, and tubes were rinsed with PBS until runoff was colorless. Finally, biofilms were dissolved in 95% ethanol for 15 min, and absorbance at 595 nm was measured using a UV-VIS spectrophotometer to determine MBIC and MBEC. The untreated sample contained only the growth medium, sterile distilled water with 0.5% Tween 80, and yeast cell suspension. The negative control contained only the growth medium. The experiment was repeated four times, and average values were obtained.

### 2.9 Statistical analysis

We used Dixon’s Q test to identify and remove outliers when analyzing microbial growth or biofilm assay data. For each concentration tested in the assays mentioned above, we calculated the arithmetic mean and standard deviation (SD), which were presented as a relative percentage compared to the control sample (set at 100%). Dixon’s Q test was utilized to identify outliers in the data obtained from the biofilm assays (Kulišová et al., 2024). We performed a one-way analysis of variance (ANOVA) with a significance level of *p* < 0.05 to assess the statistical significance of the disparities observed between the control and adjuvant effects (Krueger et al., 2024). Statistical analysis was conducted using standard protocols for microbial diversity and biofilm formation studies (Morales-Medina, 2021). The approval of an ethics committee is not necessary for this study.

## 3 Results

### 3.1 Microscopic morphology

**FIGURE 1:**
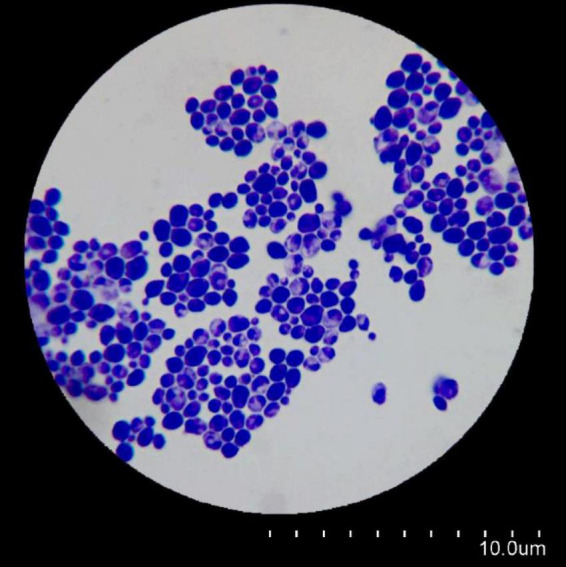
Microscopic morphology of *Candida albicans* using a simple staining method.

*C. albicans* fungal cells were uniformly observed in a yeast form (Figure 1).

### 3.2 Investigation of the antifungal activity of TTO against *C. albicans*

**FIGURE 2:**
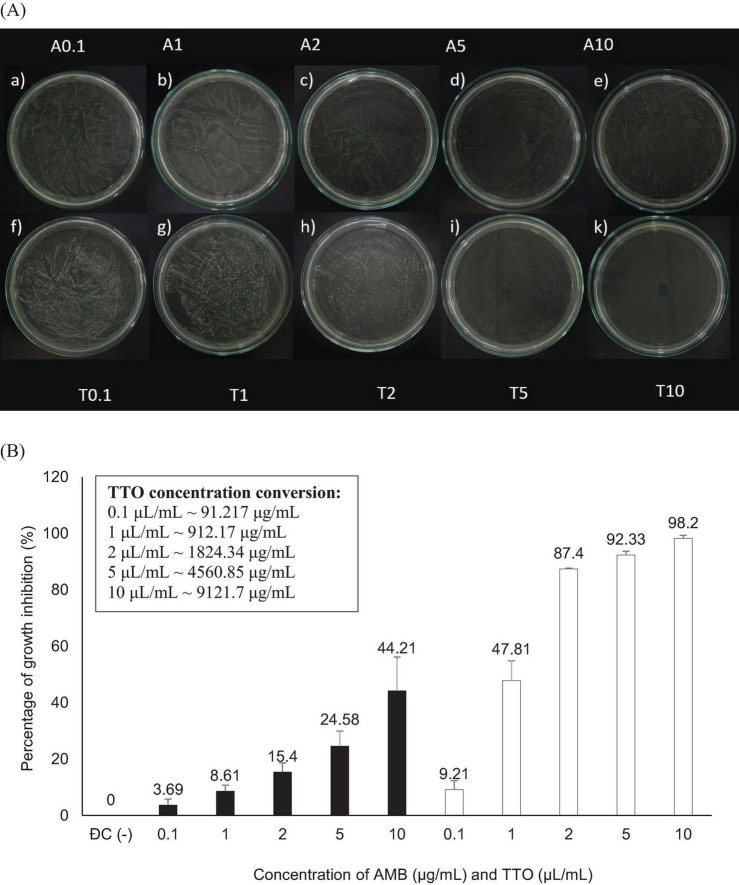
Antifungal activity against *Candida albicans* of the antifungal agent AMB and TTO at various test concentrations. **(A)** MFC results are presented as follows: Images labeled a), b), c), d), e) show samples treated with the antifungal agent AMB at concentrations of 0.1, 1, 2, 5, and 10 μg/mL, respectively; and f), g), h), i), k) display samples treated with TTO at concentrations of 0.1, 1, 2, 5, and 10 μL/mL (equivalent to approximately 91.217, 912.17, 1824.34, 4560.85, and 9121.7 μg/mL, respectively) respectively. **(B)** Graphical representation of concentration thresholds inhibiting fungal growth.

The results determined by two criteria, MIC and MFC, are presented in Figure 2 and Table 1.

**TABLE 1:** MIC and MFC of the AMB antifungal agent and TTO.

Bioactive compounds	MIC (Mean ± SD)	MIC_80_ (Mean ± SD)	MFC (Mean ± SD)
AMB	0.1 μg/mL (3.69 ± 2.09)	–	–
TTO	0.1 μL/mL (∼91.217 μg/mL) (9.21 ± 3.11)	2 μL/mL (∼1824.34 μg/mL) (87.4 ± 0.27)	10 μL/mL (∼9121.7 μg/mL) (98.2 ± 1.13)

(−): not detected. The MIC and MFC values were determined as the average of three independent experiments, and the results are presented as mean ± standard deviation (SD) to ensure reproducibility and accuracy.

Figure 2A illustrates that both the antifungal agent AMB and TTO at all tested concentrations exhibit a decrease in optical density compared to the negative control (−), indicating MIC values of 0.1 μg/mL for AMB and 0.1 μL/mL (∼91.217 μg/mL) for TTO. All concentrations of the positive control (treated with AMB) show a growth inhibition percentage in *C. albicans* not exceeding 50%. For the TTO-treated sample, the antifungal activity of the oil follows the pattern of “increasing concentration of the active substance, increasing antifungal activity,” similar to the AMB antifungal agent.

Based on the results presented in Figure 2B and Table 1, at a concentration of 2 μL/mL (∼1824.34 μg/mL), the TTO-treated sample caused a reduction of over 80% in optical density compared to the negative control, specifically inhibiting the growth of *C. albicans* by 87.4 ± 0.27%. This concentration is noted as MIC_80_. The survey results also recorded the MIC of tea tree oil against *C. albicans* as 0.1 μL/mL (∼91.217 μg/mL). Regarding the MFC criterion, only the sample treated with TTO at a concentration of 10 μL/mL (∼9121.7 μg/mL) showed no formation of fungal clusters on the surface of the Hansen medium after 48 h of incubation.

To further elucidate the antifungal activity of TTO, the MFC/MIC ratio was calculated to classify its effect as fungistatic (MFC/MIC > 4), fungicidal (MFC/MIC ≤ 4), or tolerant. Based on the data, TTO exhibited a fungicidal activity against *Candida albicans*, with an MFC/MIC ratio of ≤4 in all tested conditions. This ratio provides critical insights into the mechanism of action of TTO, distinguishing it from agents with fungistatic properties.

### 3.3 Investigation of the biofilm formation capability of *C. albicans*

The experiment was conducted with three independent trials, and each trial included three parallel samples. The results of the experiment are shown in Tables 2, 3 and Supplementary Figure 1.

**TABLE 2:** The mean ODs value at a wavelength of 570 nm.

Trial	Symbol	The mean ODs value at a wavelength of 570 nm	ODc value
Hansen + 2.5% glucose	NT1	2.6390 ± 1.6245	1.3127 ± 0.3875
Hansen + 5% glucose	NT2	2.4337 ± 1.5600	
Hansen + 10% glucose	NT3	2.2874 ± 1.5124	

When comparing, the ODc value is taken to approximately two decimal places, ODc = 1.31.

**TABLE 3:** The extent of biofilm formation by *Candida albicans* in the Hansen medium supplemented with varying percentages of glucose (w/v).

The extent of biofilm formation	Trials		
	**Hansen + 2.5% glucose** **(NT1)**	**Hansen + 5% glucose** **(NT2)**	**Hansen + 10% glucose** **(NT3)**
No biofilm producer ODs ± 1.31	**–**	**–**	**–**
Weak biofilm producer 1.31 ± ODs ± 2.62	**–**	**+**	**+**
Moderate biofilm producer 2.62 ± ODs ± 5.24	**+**	**–**	**–**
Strong biofilm producer 5.24 ± ODs	**–**	**–**	**–**

(+): biofilm formation at the corresponding level; (−): no biofilm formation at the corresponding level.

According to the data presented in Tables 2, 3, all three experimental trials indicate that the addition of varying percentages of glucose (w/v) to the Hansen medium can stimulate *C. albicans* to form biofilm (refer to Table 3). Analyzing the results from Table 3, NT1 exhibits the most proficient biofilm-forming capacity of *C. albicans*, achieving a moderate level of biofilm formation, in contrast to the other two trials which demonstrate weaker biofilm formation. This disparity could potentially be attributed to the elevated levels of supplemental D-glucose in the Hansen medium at 5 and 10%, inadvertently acting as an inhibitory factor that impedes biofilm formation. Consequently, there is an observable tendency toward decreased biofilm formation under these conditions.

**FIGURE 3:**
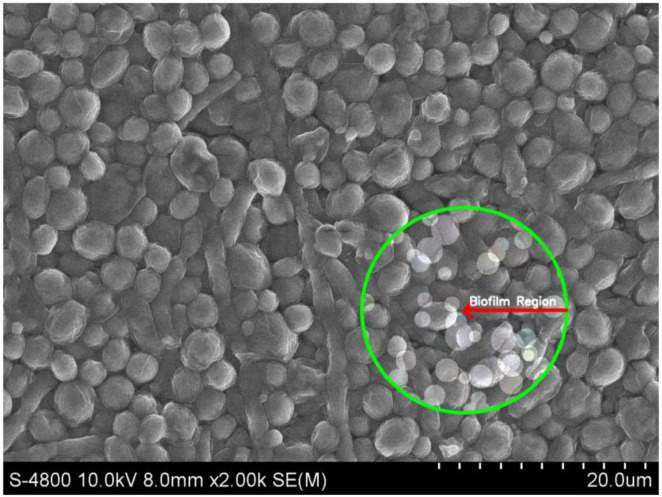
Scanning Electron Microscopy (SEM) image of *Candida albicans* biofilm in the experimental condition of Hansen medium + 2.5% glucose. The marked region (green circle) highlights the biofilm formation.

Biofilm formation by *C. albicans* on Hansen medium supplemented with 2.5% glucose was documented through electron microscopy (Figure 3). Consequently, the Hansen medium enriched with 2.5% glucose is characterized as having the capacity to foster a moderate level of biofilm formation, establishing a foundation for subsequent investigations.

### 3.4 Exploring the inhibitory impact of TTO on *C. albicans* biofilm formation

**FIGURE 4:**
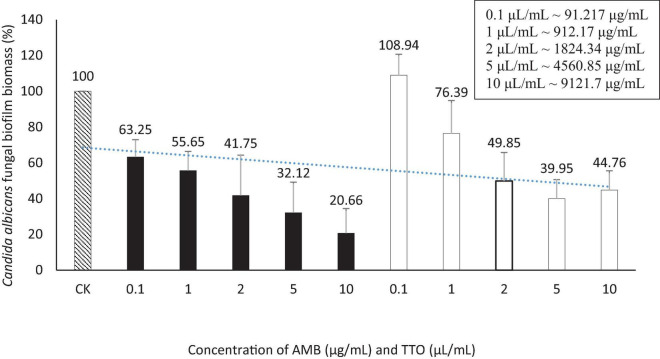
Suppression of *Candida albicans* biofilm formation by AMB and TTO at different investigated concentrations (following MBIC). For AMB, statistically significant differences (*p* < 0.05) compared to the control included concentrations (μg/mL) of 0.1, 1, 2, 5, and 10; For TTO, statistically significant differences (*p* < 0.05) included concentrations (μL/mL) of 2, 5, and 10 (equivalent to approximately 1824.34, 4560.85, and 9121.7 μg/mL, respectively).

**FIGURE 5:**
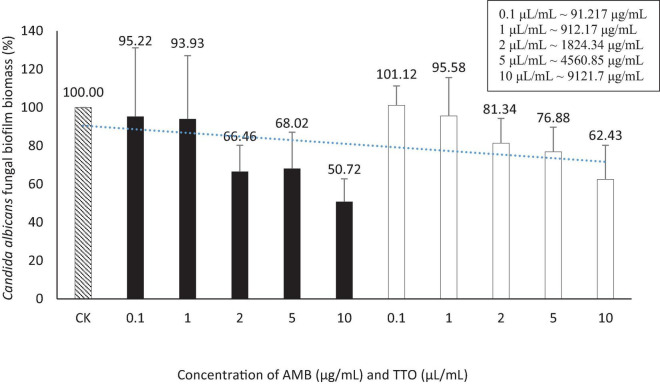
Eradication of *Candida albicans* biofilm formation by AMB and TTO at different test concentrations (following MBEC). For AMB, the statistically significant difference (*p* < 0.05) compared to the control had a threshold of only 10; For TTO, all concentrations were not statistically significant (*p* > 0.05).

The outcomes presented above provide the basis for the selection of an optimal biofilm-forming environment for *C. albicans* in this experiment. Consequently, the “Hansen medium supplemented with 2.5% glucose (w/v)” has been chosen as the experimental condition for biofilm formation. This choice is informed by its superior biofilm-forming capability compared to the other conditions investigated. The trial was carried out concurrently with four parallel samples. The outcomes were assessed using two metrics: MBIC, presented in Figure 4 and Table 4, and MBEC, as demonstrated in Figure 5 and Table 4.

**TABLE 4:** The MBIC and MBEC values for the antifungal agent AMB and TTO were determined in this study

Bioactive compounds	MBIC (mean ± SD)	MBEC (mean ± SD)
AMB	2 μg/mL (41.75 ± 22.54)	—
TTO	2 μL/mL (∼1824.34 μg/mL) (49.85 ± 15.91)	—

(–): not detected. The MBIC and MBEC values were determined as the average of four independent experiments, and the results are presented as mean ± standard deviation (SD) to ensure reproducibility and accuracy.

We performed *t*-tests to compare the inhibitory effects of AMB and TTO (following MBIC) on *C. albicans* against the control (CK) at different concentrations. The statistical analysis revealed significant differences between the control and various concentrations of both substances. For AMB, a significant inhibitory effect was observed as early as 0.1 μg/mL (p ∼ 0.022), with the significance increasing at higher concentrations, and a highly significant difference at 10 μL/mL (p ∼ 0.010). Similarly, for TTO, starting at 2 μL/mL (∼1824.34 μg/mL), there was a statistically significant difference compared to the control (*p* < 0.05). At 5 μL/mL (∼4560.85 μg/mL) (p ∼ 0.010) and 10 μL/mL (∼9121.7 μg/mL) (p ∼ 0.012), the difference was highly significant (*p* < 0.01), indicating a strong inhibitory effect at higher concentrations. These results demonstrate that both AMB and TTO exhibit substantial antifungal activity against *C. albicans*, particularly at higher concentrations (see Supplementary Table 1).

The statistical analysis using *t*-tests to compare the inhibitory effects of AMB and TTO (following MBEC) compared to the control at different concentrations on *C. albicans* revealed varying degrees of effectiveness at different concentrations. For AMB, there were no significant differences at the concentrations of 0.1 and 1 μg/mL (*p* > 0.05), indicating no notable inhibitory effect at these lower concentrations. At 2 μg/mL, although the difference was not statistically significant (p ∼ 0.052), the result was close to significance, suggesting that inhibitory effects might begin to manifest at this concentration. The concentration of 5 μg/mL also did not show a significant difference (p ∼ 0.101), but at the highest concentration of 10 μg/mL, a statistically significant difference was observed (p ∼ 0.019), indicating a strong inhibitory effect. For TTO, the concentrations of 0.1 μL/mL (∼91.217 μg/mL) and 1 μL/mL (∼912.17 μg/mL) did not result in significant differences (*p* > 0.05), suggesting no strong inhibitory effect at these levels. At 2 μL/mL (∼1824.34 μg/mL), although no significant difference was observed (p ∼ 0.130), the data indicate that inhibitory effects might start appearing. The result for 5 μL/mL (∼4560.85 μg/mL) was close to significance (p ∼ 0.089), while at 10 μL/mL (∼9121.7 μg/mL), although not statistically significant, the difference approached significance (p ∼ 0.067), suggesting a considerable inhibitory potential at this higher concentration. In summary, AMB demonstrated a significant inhibitory effect at 10 μg/mL, and while TTO did not reach statistical significance at 10 μL/mL (∼9121.7 μg/mL), the data suggest a notable inhibitory effect at this concentration (see Supplementary Table 2).

Regarding the MBEC, the findings depicted in Figure 5 reveal an incremental trend in the eradication capacity of pre-formed *C. albicans* biofilm with rising concentrations of the bioactive agents (AMB and TTO). However, neither AMB nor TTO exhibited a specific MBEC value, as the biofilm mass at all tested concentrations surpassed 50% when compared to the untreated control. In essence, the biofilm eradication efficacy of both bioactive compounds remained below 50%, precluding the determination of a definitive MBEC value. At a concentration of 10 μg/mL, AMB eradicated only 49.28% of the *C. albicans* biofilm mass, while for TTO treatment at the highest concentration of 10 μL/mL, the reduction in biofilm mass after 24 h of pre-culture was recorded at 62.43 ± 17.82% compared to the untreated control.

## 4 Discussion

This study provides a comprehensive evaluation of the antifungal and antibiofilm potential of Tea Tree Oil (TTO) derived from *Melaleuca alternifolia* against *Candida albicans*. Key findings reveal that TTO demonstrates significant antifungal activity, with a Minimum Inhibitory Concentration (MIC) of 0.1 μL/mL (∼91.217 μg/mL) and a Minimum Fungicidal Concentration (MFC) of 10 μL/mL (∼9121.7 μg/mL), indicating fungicidal properties supported by an MFC/MIC ratio of ≤4. Additionally, TTO effectively inhibits biofilm formation at a Minimum Biofilm Inhibitory Concentration (MBIC) of 2 μL/mL (∼1824.34 μg/mL). However, the eradication of mature biofilms was only partial, with no definitive Minimum Biofilm Eradication Concentration (MBEC) observed at the tested concentrations. Gas chromatography-mass spectrometry (GC-MS) analysis highlights the presence of bioactive components, particularly terpinen-4-ol and α-terpineol, which likely contribute to TTO’s antifungal efficacy. These findings underscore the potential of TTO as a natural antifungal agent, offering new insights into its application against biofilm-associated fungal infections while identifying challenges, such as its limited biofilm-eradication capability.

### 4.1 Investigation of the antifungal activity against *C. albicans* of TTO

The experiments demonstrated that adding sterile distilled water containing 0.5% Tween 80 to Mueller-Hinton medium promotes the growth of *C. albicans*, as opposed to the medium without this supplementation. This observation is consistent with the findings of Carson and Riley, who suggested that Tween 80 serves as a stimulant due to its oleic acid content (Carson and Riley, 1994). Consequently, the Mueller-Hinton medium supplemented with sterile distilled water containing 0.5% Tween 80 is designated as the negative control for comparison with samples treated with the antifungal agent AMB and TTO.

The MIC of TTO against *C. albicans* was found to be 0.1 μL/mL (∼91.217 μg/mL) in our study. However, other studies, such as Thosar et al. (2013), reported a MIC of 0.5 μL/mL (∼456.085 μg/mL) for *C. albicans*. Rosato et al. (2008) demonstrated MIC values ranging from 1.75 to 3.5 mg/mL for various *Candida* strains treated with TTO. In contrast, Oro et al. (2015) reported a lower average MIC of 20.02 μg/mL for *C. albicans* when exposed to TTO. These variations suggest differing sensitivities among *C. albicans* strains.

MFC values for TTO against *C. albicans* exhibit variability among studies. Oro et al. (2015) reported an MFC of 25.33 μg/mL, while Francisconi et al. (2015) found it to be 0.25% TTO. Noumi et al. (2011) observed different MFC values across *C. albicans* strains, ranging from 5 to >10 mg/mL. This variation highlights the diverse sensitivity of *C. albicans* strains to TTO, mirroring trends seen in MIC values.

In terms of mechanism, AMB acts by creating pores in the fungal cell membrane, altering permeability, and causing cell death. TTO is thought to penetrate the cell wall and plasma membrane of *C. albicans*, disrupting these structures and leading to cell leakage due to its lipophilic nature.

TTO analysis identified eucalyptol (or 1,8-cineole) as the predominant component, comprising 29.41% of the oil. Other notable constituents include terpinolene (8.63%), γ-terpinene (7.92%), α-terpineol (5.67%), and various trace components. The antifungal efficacy of TTO is attributed to the presence of 1,8-cineole, which has been shown in studies to enhance fungal cell membrane permeability (Brun et al., 2019; Hammer et al., 2003a). This facilitates the penetration of other oil components into the cells, influencing internal cellular processes (Brun et al., 2019).

### 4.2 Comparison with previous TTO formulations

The antimicrobial activity of TTO against *C. albicans* and its ability to inhibit biofilm formation have been extensively studied. However, our study utilizes a modified TTO formulation with altered concentrations of key active components, such as terpinen-4-ol and α-terpineol. These components are known to contribute significantly to the antimicrobial efficacy of TTO. For instance, previous research has shown that terpinen-4-ol, one of the major constituents of TTO, plays a crucial role in the inhibition of fungal growth and biofilm formation (Hammer et al., 2003a). Higher concentrations of terpinen-4-ol in TTO formulations have been associated with stronger antifungal activity, while formulations with lower concentrations of this component demonstrate reduced efficacy (Carson et al., 2006). In our formulation, the increased concentration of terpinen-4-ol likely contributes to the enhanced biofilm inhibition observed in our study compared to other formulations.

### 4.3 Impact of specific components on antimicrobial activity

In addition to terpinen-4-ol, α-terpineol is another important component of TTO that contributes to its antimicrobial properties. It has been reported that α-terpineol enhances the overall antifungal effects of TTO by increasing the disruption of microbial cell membranes (Mondello et al., 2006). The synergistic effects of terpinen-4-ol and α-terpineol may explain the high efficacy of our modified TTO formulation in inhibiting *C. albicans* biofilm.

### 4.4 Toxicity considerations

Although this study focuses primarily on the antimicrobial effects of TTO, the potential toxicity of its components should also be considered. Research has shown that terpinen-4-ol and α-terpineol can exhibit cytotoxic effects at high concentrations, particularly in skin and mucosal tissues (Hammer et al., 2002). However, these cytotoxic effects tend to occur at concentrations higher than those used in typical antimicrobial applications (Cox et al., 2001). While we did not directly assess the toxicity of our modified TTO formulation, future studies would be necessary to evaluate its safety, especially for therapeutic applications.

### 4.5 Investigation of *C. albicans* biofilm formation

The study conducted by Brambilla et al. (2016) revealed an increased biofilm formation of *C. albicans* with the rising percentage of D-glucose in the Trypticase-Soy broth (TSB) medium, ranging from 1.25 to 10% (w/v). This finding contrasts with the survey results, given the varying glucose concentrations in the growth media used for *C. albicans* biofilm formation. Specifically, TSB medium contains 2.5 g/L of glucose, while Hansen medium boasts a glucose concentration of up to 50 g/L. Consequently, this discrepancy may have impacted *C. albicans’* biofilm formation in the study, leading to a tendency of reduced biofilm formation in NT2 and NT3 due to the exceptionally high sugar content in the experimental environment, inadvertently resulting in inhibitory effects on *C. albicans*.

### 4.6 Investigating the inhibition of *C. albicans* biofilm formation by TTO

The study found a MBIC of the antifungal agent AMB at 2 μg/mL (Figure 4 and Supplementary Figure 2). Mahmoudabadi et al. (2014) investigated MBIC on 120 *C. albicans* strains, revealing that 65% of them had an MBIC below 10 μg/mL, indicating the overall sensitivity of *C. albicans* strains to AMB. The observed MBIC of 2 μg/mL aligns with the known effectiveness of AMB in inhibiting *C. albicans* biofilm formation under *in vitro* conditions (Mahmoudabadi et al., 2014).

For the TTO-treated sample, the investigation identified an MBIC value of 2 μL/mL (∼1824.34 μg/mL) (Figure 4 and Supplementary Figure 2). The diverse terpenes present in tea tree oil modify cell membrane permeability by infiltrating between the fatty-acyl chains of the cell membrane bilayer. This induces changes in membrane permeability, leading to alterations in cell surface and morphology and diminishing adhesion capabilities to *C. albicans’* substrate (Dalleau et al., 2008). This contributes to the observed reduction in biofilm formation of *C. albicans* when treated with TTO.

The absence of recorded MBEC values for both biological agents (AMB and TTO) in the survey (Table 4) may be attributed to the tea tree oil concentration examined not being sufficiently high to disrupt more than 50% of the pre-cultured biofilm mass within 24 h. At the highest concentration of tea tree oil in the survey (10 ∼ 1824.34 μg/mL), it was observed to disrupt 37.57% of the *C. albicans* biofilm mass compared to the untreated sample, as indicated in Figure 5.

In our study, we acknowledge several limitations that could impact the interpretation of the findings. Firstly, although the antifungal activity of Tea Tree Oil (TTO) against *Candida albicans* and its biofilm formation inhibition has been previously studied, our research presents a modified TTO formulation with an increased concentration of terpinen-4-ol, aiming to enhance its antifungal efficacy and biofilm inhibition. This formulation provides a novel perspective on the role of terpinen-4-ol and α-terpineol synergy, which has not been extensively evaluated. Secondly, we focused on a single strain of *C. albicans* (ATCC 24433), which limits the generalizability of our findings. Future research should include a diverse set of clinical isolates, including other pathogenic *Candida* species, to better assess the broad-spectrum efficacy of the formulation. Additionally, we employed standard biofilm quantification methods like crystal violet staining. Incorporating more robust techniques such as CFU counts or metabolic assays in future studies would strengthen the findings. Furthermore, we explored only one TTO formulation; comparisons with other TTO formulations or isolated components (e.g., terpinen-4-ol) could provide a more comprehensive understanding of its antifungal properties. Lastly, while this study focuses on the antifungal and biofilm-inhibitory potential of TTO, we did not investigate its synergistic effects with conventional antifungal agents, which could offer enhanced therapeutic strategies. These elements, when addressed in future studies, could significantly elevate the impact and relevance of our findings.

The MFC determination in this study was performed using a protocol adapted from Rodriguez-Tudela et al. (2001), as no universal standard for fungal MFC testing currently exists. While this method is widely accepted for antifungal research, its limitations must be acknowledged. The use of 50 μL subcultures may not capture the ≥99.9% reduction in viable fungal cells defined for bactericidal agents by CLSI. Nevertheless, this approach provides a reproducible and practical means to assess fungicidal activity in *Candida albicans*, aligning with established methodologies for fungal studies. Future research should aim to standardize MFC determination in yeasts to ensure consistency and comparability across studies.

## 5 Conclusion

In this study, we investigated the antifungal efficacy of a modified formulation of TTO against *C. albicans*, focusing on both its planktonic and biofilm states. Our findings establish the MIC of TTO at 0.1 μL/mL (∼91.217 μg/mL) and the MFC at 10 μL/mL (∼9121.7 μg/mL), demonstrating its strong antifungal activity. Notably, we identified a MBIC of 2 μL/mL (∼1824.34 μg/mL), highlighting TTO’s potent ability to inhibit biofilm formation. This study is among the first to provide a comprehensive evaluation of TTO’s effects on *C. albicans*, particularly in the context of biofilm formation, which is a critical factor in persistent fungal infections.

The modified TTO formulation used in this study, characterized by increased concentrations of terpinen-4-ol and α-terpineol, exhibited enhanced antifungal and biofilm inhibitory properties compared to previously studied formulations. These findings underscore the potential of this modified TTO formulation as a novel therapeutic agent for managing *C. albicans* infections, particularly those associated with biofilm formation. Further research into the toxicity profile and clinical applications of this formulation is warranted to fully explore its potential as a natural antifungal treatment.

## Data Availability

The original contributions presented in this study are included in this article/Supplementary material, further inquiries can be directed to the corresponding author.
