# Supplementary material for: Innovative antifungal strategies: enhanced biofilm inhibition of Candida albicans by a modified tea tree oil formulation
Source: Front Microbiol. 2025 Jan 15;15:1518598. doi: 10.3389/fmicb.2024.1518598 (PMC11778174; doi:10.3389/fmicb.2024.1518598)
Supplement: Supplementary file 1 [file Data_Sheet_1.docx]

**Table S1: Statistical Analysis of t-test Results for Amphotericin B (AMB) and Tea Tree Oil (TTO) Inhibitory Effects on Candida albicans (following MBIC)**

| **Sample** | **Concentration** | **t-stat** | **p-value** |
| --- | --- | --- | --- |
| AMB (μg/mL) | 0.1 | 6.58251 | 0.02231 |
|  | 1 | 7.172405 | 0.01889 |
|  | 2 | 4.47613 | 0.04646 |
|  | 5 | 6.887616 | 0.02044 |
|  | 10 | 9.958037 | 0.00993 |
| TTO (μL/mL) | 0.1 | -1.32233 | 0.31702 |
|  | 1 | 2.222485 | 0.15632 |
|  | 2 | 5.459607 | 0.03195 |
|  | 5 | 9.757003 | 0.01034 |
|  | 10 | 8.86733 | 0.01248 |

**Table S2: Statistical Analysis of t-test Results for Amphotericin B (AMB) and Tea Tree Oil (TTO) Inhibitory Effects on Candida albicans (following MBEC)**

| **Sample** | **Concentration** | **t-stat** | **p-value** |
| --- | --- | --- | --- |
| AMB (μg/mL) | 0.1 | 0.23017 | 0.839359 |
|  | 1 | 0.317055 | 0.781238 |
|  | 2 | 4.206588 | 0.052132 |
|  | 5 | 2.901571 | 0.101087 |
|  | 10 | 7.107033 | 0.019229 |
| TTO (μL/mL) | 0.1 | -0.1915 | 0.865814 |
|  | 1 | 0.382019 | 0.739219 |
|  | 2 | 2.49191 | 0.130297 |
|  | 5 | 3.116344 | 0.08938 |
|  | 10 | 3.651692 | 0.067488 |

**Table S3: TTO essential oil composition analysis results**

| **TT** | **Rt** | **Components** | **Mass** | **Mass spectrum match** | **Content (%)** |
| --- | --- | --- | --- | --- | --- |
| 1 | 6.86 | α-Thujene | 136 | 926 | 1.20 |
| 2 | 7.08 | 1R-. α.-Pinene | 136 | 941 | 2.41 |
| 3 | 8.86 | . β.-Pinene | 136 | 932 | 0.72 |
| 4 | 10.25 | α-Phellandrene | 136 | 923 | 0.79 |
| 5 | 10.89 | α.-Terpinene | 136 | 902 | 1.51 |
| 6 | 11.32 | β-Cymene | 134 | 933 | 2.36 |
| 7 | 11.52 | D-Limonene | 136 | 932 | 2.43 |
| 8 | 11.64 | Eucalyptol | 154 | 969 | 29.41 |
| 9 | 13.23 | γ-Terpinene | 136 | 963 | 7.92 |
| 10 | 14.95 | .Terpinolene | 136 | 957 | 8.63 |
| 11 | 15.86 | Linalool | 154 | 849 | 1.30 |
| 12 | 20.03 | .4-Terpineol | 154 | 866 | 0.82 |
| 13 | 20.70 | .. α-Terpineol | 154 | 920 | 5.67 |
| 14 | 28.89 | Caryophyllene | 204 | 948 | 5.21 |
| 15 | 29.89 | Humulene | 204 | 912 | 3.32 |
| 16 | 30.55 | Unknown | - | - | 0.93 |
| 17 | 30.67 | α-Amorphene | 204 | 911 | 0.84 |
| 18 | 30.83 | β-Eudesmene | 204 | 946 | 2.36 |
| 19 | 30.97 | δ-Selinene | 204 | 887 | 0.92 |
| 20 | 31.08 | α-Selinene | 204 | 929 | 2.48 |
| 21 | 31.41 | β-Cadinene | 204 | 890 | 1.01 |
| 22 | 33.43 | Guaiol | 222 | 917 | 4.69 |
| 23 | 34.07 | .γ-Eudesmol | 222 | 901 | 3.59 |
| 24 | 34.41 | β-Eudesmol | 222 | 907 | 3.28 |
| 25 | 34.46 | α-Eudesmol | 222 | 915 | 4.73 |
| 26 | 34.69 | Bulnesol | 222 | 889 | 1.51 |

- **Analytical index:** Analysis of the composition and relative content of compounds in the Tea Tree essential oil sample using GCMS method.
- Essential oil sample (25 µL) dissolved in 1.0 mL n-hexane. Sample injection: 1.0 µL, split ratio 1:50
- GC Agilent 6890N, MS 5973 inert. HP5-MS column, He pressure at the top of the column 9.3 psi
- Temperature program for the sample: 50 ^o^C held for 2 minutes then increased by 2 ^o^C/min to 80 ^o^C, increased by 5 ^o^C/min to 150 ^o^C, continued to increase by 3 ^o^C/min to 180 ^o^C, increased by 20 ^o^C/min to 300 ^o^C held for 5 minutes.

| 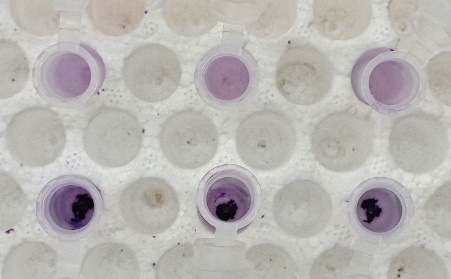  (a) | 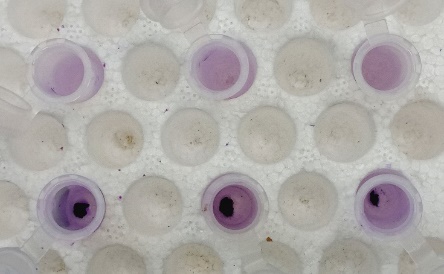  (b) | 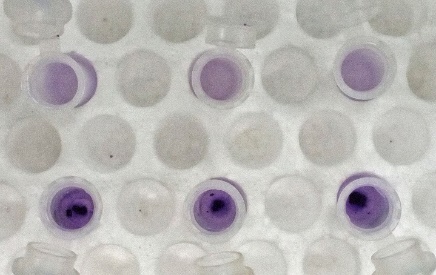  (c) |
| --- | --- | --- |

**Figure S1.** **Biofilm image of *Candida albicans* stained with 1% crystal violet in Hansen medium was supplemented with an additional percentage (w/v) of glucose**. (a) Hansen + 2.5% glucose; (b) Hansen + 5% glucose; (c) Hansen + 10% glucose.

**
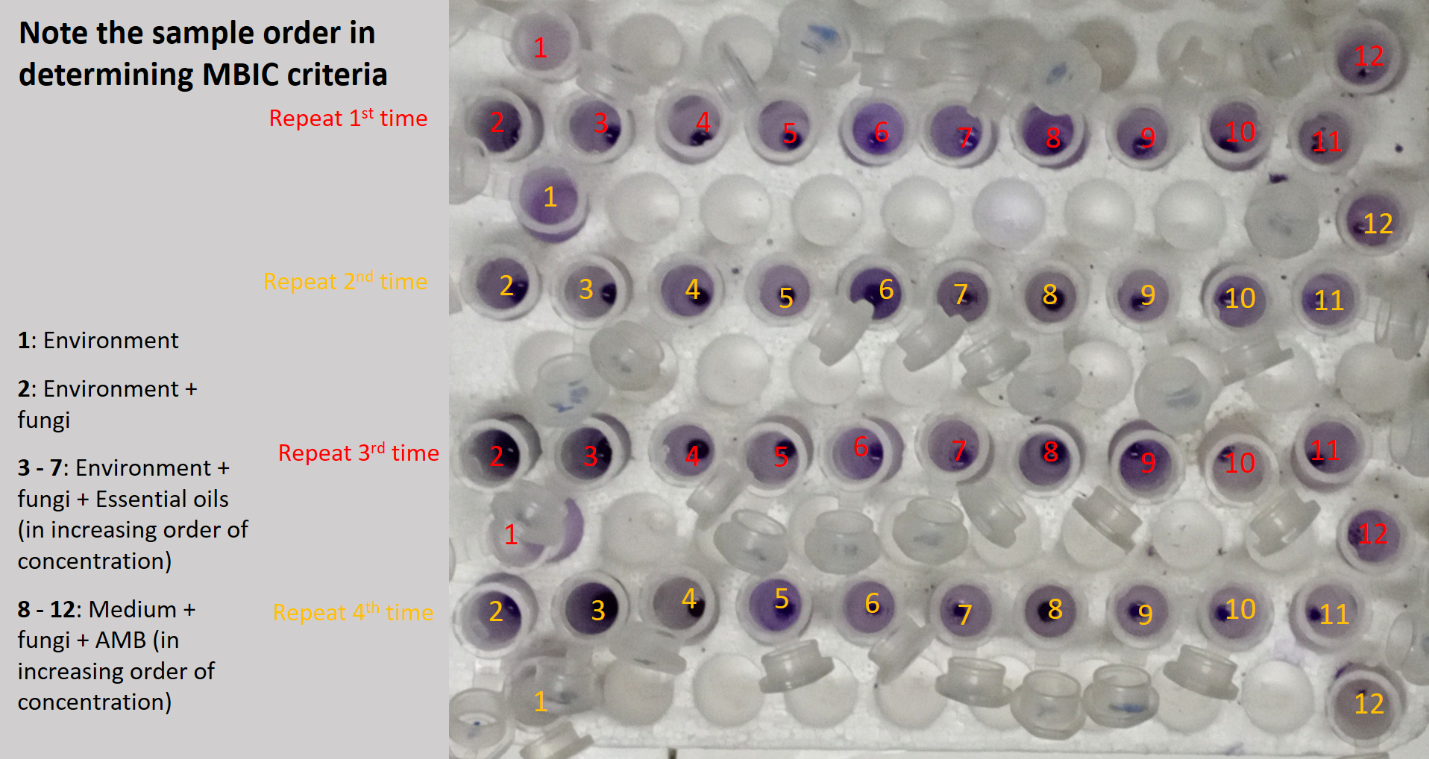
**

**
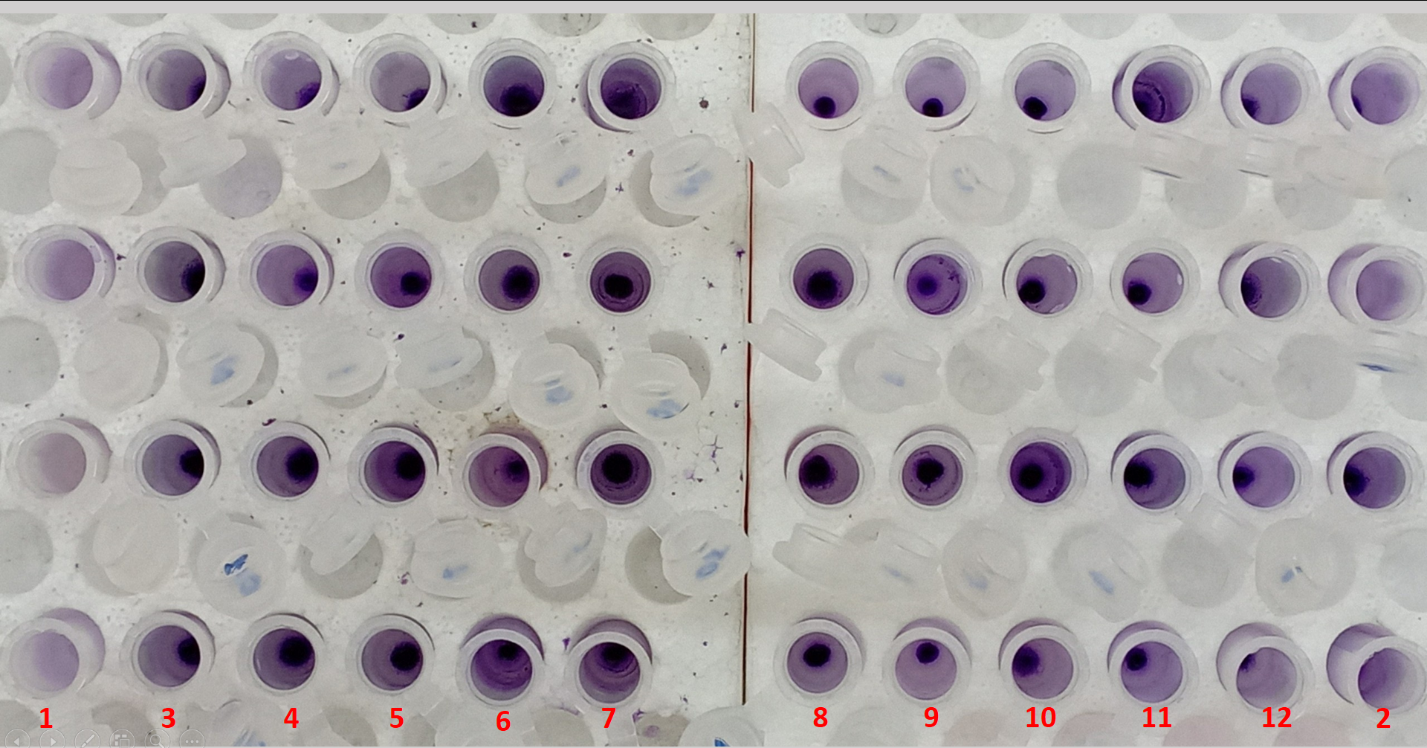
**

**Figure S2. Biofilm image of *Candida albicans* to determine MBIC and MBEC**. (a) MBIC; (b) MBEC (the note-taking is similar to the MBIC).
